# Ostm1 from Mouse to Human: Insights into Osteoclast Maturation

**DOI:** 10.3390/ijms21165600

**Published:** 2020-08-05

**Authors:** Jean Vacher, Michael Bruccoleri, Monica Pata

**Affiliations:** 1 Institut de Recherches Cliniques de Montreal (IRCM), Montreal, QC H2W 1R7, Canada; michael.bruccoleri@ircm.qc.ca (M.B.); patam@ircm.qc.ca (M.P.); 2 Departement de Medecine, Universite de Montreal, Montreal, QC H2W 1R7, Canada; 3 Department of Medicine, Division of Experimental Medicine, McGill University, Montreal, QC H3A 1A3, Canada

**Keywords:** osteoclast, osteopetrosis, grey-lethal, Ostm1, bone resorption, trafficking

## Abstract

The maintenance of bone mass is a dynamic process that requires a strict balance between bone formation and resorption. Bone formation is controlled by osteoblasts, while osteoclasts are responsible for resorption of the bone matrix. The opposite functions of these cell types have to be tightly regulated not only during normal bone development, but also during adult life, to maintain serum calcium homeostasis and sustain bone integrity to prevent bone fractures. Disruption of the control of bone synthesis or resorption can lead to an over accumulation of bone tissue in osteopetrosis or conversely to a net depletion of the bone mass in osteoporosis. Moreover, high levels of bone resorption with focal bone formation can cause Paget’s disease. Here, we summarize the steps toward isolation and characterization of the osteopetrosis associated trans-membrane protein 1 (*Ostm1*) gene and protein, essential for proper osteoclast maturation, and responsible when mutated for the most severe form of osteopetrosis in mice and humans.

## 1. Introduction

Osteoclasts derive from hematopoietic stem cells that are shared with early myeloid lineage precursors. Differentiation of osteoclast precursors is dependent on mature osteoblasts that produce macrophage colony-stimulating factor (M-CSF), receptor activator of NF-κB Ligand (RANKL), and osteoprotegerin (OPG) a soluble decoy receptor of RANKL [1,2,3,4,5]. Upon recruitment and attachment to bone, mononuclear pre-osteoclasts undergo a process of fusion and these newly-formed multinucleated cells are structurally and functionally induced to generate active osteoclasts [6]. Mature osteoclasts are large multinucleated cells with numerous mitochondria, vacuoles, and lysosomes, which resorb mineralized cartilage and bone [7].

The biochemical characterization of osteoclasts have been hampered by the fact that these giant cells are tightly attached to the bone matrix and are therefore difficult to isolate. Moreover, as these cells are terminally differentiated and non-proliferative, a large number of cells have to be isolated at once. However, despite these impediments, osteoclast specific markers have been defined and novel efficient tools have been developed to analyze osteoclast biology ex vivo and in vivo [8].

When osteoclasts are activated, a resorption cycle is induced causing several proteins to be relocalized along with cytoskeletal rearrangement. Active osteoclasts are polarized and show two cellular histo-morphologic characteristics: an actin ring and a ruffled border. The actin ring, devoided of organelles, is enriched in dynamic and adhesive projections of the cell membrane called podosomes and in αVβ3 integrins that allow spreading and tight attachment to the bone surface [9,10,11]. The plasma membrane in contact with the bone surface enlarges into the ruffled border that induces polarization of the osteoclast. Following this attachment, the osteoclast secretory lysosomes, also found in immune cells and melanocytes [12,13], will associate and move along the microtubules, fuse to the plasma membrane, and then participate in ruffled border formation [14,15]. The ruffled border is an infolded finger-like distortion of the plasma membrane adjacent to the bone surface that participates with lysosomal proton pump H^+^/V-ATPase and chloride exchanger ClC-7 in acidification of the extracellular resorbing lacunae to ensure bone matrix demineralization [16,17,18]. In the lacunae, release from secretory lysosomes of tartrate resistant acid phosphatase (Trap), matrix metallopeptidases (Mmp 9,14) [19], and cathepsin K (Ctsk) result in osteoid degradation which is principally type I collagen [20] whereas high acidity potentiates dissolution of hydroxyapatite, the bone mineral component. The protein and mineral degradation products are phagocytosed at the ruffled border into of the osteoclast as digestive vacuole. Thus, bone resorption involves exocytosis and endocytosis at the ruffled border and exocytosis on the contralateral side of osteoclasts [10,21]. Importantly, osteoclast bone resorption has been demonstrated to be critical for normal hematopoietic progenitors recruitment and proliferation that link bone remodeling to hematopoiesis regulation [22]. Furthermore, numerous fundamental bone–immune interactions through shared factors have been discovered and are the subject of the field of osteoimmunology [23,24,25,26].

Defective osteoclast differentiation or generation of inefficient osteoclasts leads to the severe bone pathology called osteopetrosis, a heterogenous inherited disease of bone metabolism [27,28]. This disease was first described by Albers-Schönberg [29] and results in accumulation of mineralized osteoid and cartilage due to loss of bone resorption [4,30]. Different forms of osteopetrosis have been characterized in various vertebrate species [31,32,33] and mouse models were essential toward our understanding of mammalian osteoclast formation and function [12,34]. In humans, three clinical groups have been defined:

- infantile-malignant autosomal recessive (ARO) which is fatal within the first few years of life;
- intermediate recessive (IRO) which appears during the first decade of life but does not mediate a malignant response;
- autosomal dominant (ADO), with full-life expectancy but with major bone malformations.

Each form of the disease is characterized by a reduced bone marrow compartment leading to hematopoietic defects including anemia and high susceptibility to infections [35,36]. Characterization of autosomal recessive osteopetrotic mutations in mouse models and in human patients defined ‘osteoclast-poor’ (impaired osteoclast differentiation) and ‘osteoclast-rich’ (inactive osteoclasts) osteopetrosis leading to more targeted therapies [37,38,39].

## 2. Osteopetrotic Grey-Lethal Mouse Model

**Figure 1:**
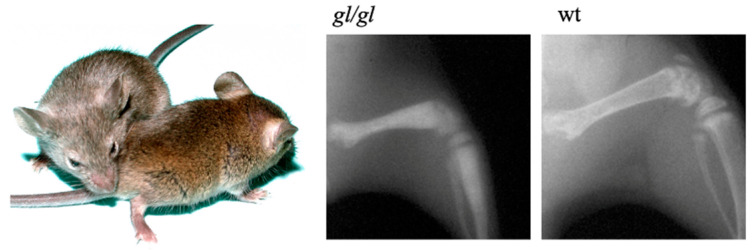
Grey coat color in *gl*/*gl* mice (left) and representative X-rays of osteopetrotic *gl*/*gl* bone compared to agouti wild-type (wt) littermate.

The spontaneous osteopetrotic grey-lethal (*gl*) mouse mutant was described by Gruneberg [40]. The homozygous *gl*/*gl* mice display a severe growth delay and a grey-coat color on an agouti background due to pheomelanin granule clumping [41]. Homozygous mice show a characteristic severe reduction of bone marrow space, lack of tooth eruption, and die around 3 weeks of age (Figure 1).

Restoration of the capacity to resorb bone matrix following normal spleen and/or bone marrow cells transplantation in *gl*/*gl* mice suggested a hematopoietic cell-intrinsic defect [42]. It is now well established that osteoclasts derived from hematopoietic precursors [43]. Our characterization of hematopoiesis in *gl*/*gl* mice was associated with mild anemia, a significant expansion of granulocyte-macrophage progenitors (CFU-GM) that give rise to osteoclasts and consistent with an increase of splenic CD11b^+^/Lys6-G^+^ monocytic cell subpopulation. In addition to this myeloid defect, deregulation of lymphoid lineages in *gl*/*gl* mice resulted in a reduction of B cell populations and altered T cell distribution with thymus hypo-cellularity [44]. This result provides the first evidence of an intrinsic time and differentiation stage-dependent molecular role for the *gl* gene in lymphoid cell lineage.

Importantly, in situ histological characterization of *gl*/*gl* bone tissue demonstrated the presence of numerous mature multinucleated osteoclasts, suggesting an intrinsic osteoclast defect that excluded cell differentiation impairment due to environmental factors. Consistent with this, ultra-structural analysis of bone sections showed that *gl*/*gl* osteoclasts are in close contact to the bone matrix but display an underdeveloped ruffled border essential for proper bone matrix resorption [45]. Accordingly, ex vivo analysis of *gl*/*gl* osteoclasts in culture demonstrated normal spreading through formation of an intact actin ring but these cells were unable to resorb bone matrix. The presence of an inactive mature osteoclast population in these mice classified the *gl*/*gl* phenotype as an ‘osteoclast-rich’ osteopetrosis [46].

## 3. Mapping the *gl* Locus and Characterization of the *Ostm1* Gene

We have successfully used a positional cloning strategy to isolate and characterize the gene responsible for the mouse osteopetrotic *gl* mutation that most closely resembles human recessive osteopetrosis. By generating—for the first time—two backcross panels penetrant for the *gl* mutation, we have produced a genetic map and reduced the genetic interval on murine chromosome 10 that included the *gl* locus from 5 cM to ~1 cM [47]. During our systematic genetic mapping of this region, identification of specific polymorphisms had a tremendous impact on the establishment of our physical and transcriptional map of 98 yeast artificial chromosomes (YAC) clones assembled in a ~8 Mb contig [48]. Additional recombination events further reduced our *gl* candidate region to a ~1000 kb genomic interval. This interval was then covered with a contig of 17 overlapping bacterial artificial chromosomes (BAC) and novel polymorphic markers narrowed our candidate interval to ~500 kb. BAC transgenic lines were produced and full functional rescue obtained with one BAC clone in transgenic homozygous *gl*/*gl* mice defined a 180kb genomic candidate segment for the localization of the *gl* locus. BAC sequencing and transcription sequence analysis defined a single gene, called *Ostm1*, which encodes a unique 3kb transcript highly expressed in osteoclasts and undetectable in homozygous *gl*/*gl* animals [49]. Of note, an additional allele of *Ostm1* (*Ostm1^om^*) was detected in an *N*-ethyl-*N*-nitrosourea (ENU) screen and results in a mild osteopetrotic phenotype but the mutation still needs to be defined [50].

*Ostm1* is widely expressed and detectable in embryonic hematopoietic, skeletal, and brain tissues which is maintained after birth along with an appearance in gut, kidney, and skin. Subsequently, full-length cDNA was isolated and we established that the *Ostm1* gene is composed of six exons and five introns. In contrast to its wild-type counterpart, *gl* genomic DNA has a ~7.5 kb deletion. This deletion covers most of the promoter, the first exon, and a large portion of the first intron leading to a null allele [49]. Importantly, targeted early re-expression of *Ostm1* in hematopoietic cells of transgenic mice with the regulatory sequences of the transcriptional factor gene PU.1 (PU.1-Ostm1) resulted in full rescue of osteopetrosis and hematopoietic defects [44]. This provided definitive evidence that *Ostm1* is the gene responsible for the *gl* mutation. Subsequently, we isolated the human *OSTM1* gene and a database search identified homologs only in metazoans. Interestingly, genomic sequence analysis of 19 osteopetrotic patients led us to characterize the first human *OSTM1* mutation associated with the disease that results in exon 5 skipping. This result was confirmed at the RNA level and allowed us to design and apply the first prenatal diagnostic test for carriers [49,51]. Additional *OSTM1* mutations with severe osteopetrosis also displayed neurological disorders [52,53] suggesting that Ostm1 activity can be essential in maintenance of neuronal cell homeostasis. In these cases, however, a secondary neuronal effect from compression of cervical nerve and foramina occlusion, due to an excess of bone, has been excluded [54,55].

In parallel, gene expression profile analyses of *gl*/*gl* hematopoietic tissue identified the Inositol polyphosphate 4-phosphatase type II (*Inpp4b*) transcript as constantly downregulated. First, we isolated and characterized the *Inpp4b* gene in the mouse [56]. Second, systemic loss of *Inpp4b* in the mouse was induced and we demonstrated that *Inpp4b* is a negative regulator of osteoclast differentiation ex vivo. These mice consistently develop an osteoporotic phenotype in vivo, linking lipid metabolism to a specific bone phenotype. In humans, we showed that specific *INPP4B* variants were associated with variable bone mineral density and established *INPP4B* as a susceptibility locus to osteoporosis in pre-menopausal women [57]. Nevertheless, while the direct link between *Ostm1* and *Inpp4b* remains to be elucidated, these results indicate that additional genomic loci can be deregulated due to the absence of *Ostm1* expression and may give rise to the discovery of novel regulators of bone mineral density in mouse and human.

## 4. Ostm1 Protein Structure and Partners

**Figure 2:**
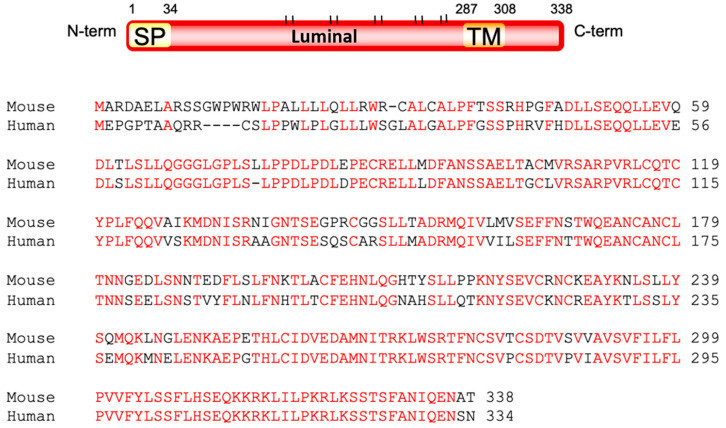
Structure of the murine Ostm1 protein and conservation between the mouse and human proteins. SP: Signal peptide; TM: Trans membrane domain; |: Glycosylation site.

The structure of the Ostm1 protein was investigated by various biochemical approaches. The open reading frame of the mouse *Ostm1* gene encodes a 338-amino acid protein while the 334 amino acid human OSTM1 protein is 83% homologous to the mouse protein. Our structural analysis defined a signal peptide and a unique trans-membrane domain that classified Ostm1 as a type I trans-membrane protein where the majority of Ostm1 is luminal with a short cytosolic 30 amino acid C-terminus (Figure 2).

Interestingly, loss of the unique transmembrane domain resulted in secretion of the protein in vitro [58,59]. The predicted mass of the mature Ostm1 protein without modifications is ~34 kDa and we established that Ostm1 protein has 10 *N*-glycosylation sites consistent with the apparent protein mass of ~60 kDa. Upon use of different inhibitors of glycosylation, we confirmed that all potential luminal sites in Ostm1 appear effectively glycosylated. This post-translational glycosylation is very rapid and occurs in the endoplasmic reticulum. Analysis of Ostm1 subcellular localization also detected Ostm1 in the Golgi apparatus and late endosome/lysosome compartment with a punctuated distribution in the cytosol [58,60].

Based on the protein structure of Ostm1, we designed a tandem affinity purification (TAP) screen using a tagged version of the C-terminus of Ostm1 [61] and identified by mass spectrometry (MS) analysis specific cytosolic partners within the EcR293 kidney cell line [58]. Interactions were validated by glutathione-s-transferase (GST) pull-down assays with the C-terminus of Ostm1 in the same cells and in RAW cell-derived osteoclasts. This screen identified proteins classified into four subgroups, several of which were confirmed to interact directly with Ostm1 by immunoprecipitation assays. This indicates that Ostm1 can have multiple interactions with cytosolic proteins and could participate in a multi-functional protein platform. Particularly, we demonstrated a direct cytosolic interaction of Ostm1 with the anterograde motor protein kinesin family member 5B (Kif5B). Co-localization experiments by live imaging showed the dynamic Ostm1/Kif5B complex re-localization and trafficking that conveyed an adaptor role for the trans-membrane Ostm1 protein. Depletion of Kif5B led to peri-nuclear clustering of Ostm1 and lysosomes, demonstrating that the Ostm1-Kif5B interaction is essential for late-endosome/lysosome organelle dispersion [58,62]. Significantly, this cellular function could elucidate some of the physiological mechanisms underlying the wide *gl*/*gl* phenotypic spectrum.

Directly related to osteoclast biology we, and others, have also detected the protein ClC-7, a Cl^−^-H^+^ exchanger, as a partner of Ostm1 [63]. The Ostm1/ClC-7 complex is localized to late endosome/lysosome membranes and is responsible for acidification of secretory lysosomes and osteoclast resorption lacunae. Similar to Ostm1, the loss of ClC-7 leads to osteopetrosis in mice and humans with neuronal defects and retinal degeneration [64,65]. However, ClC-7 null mice display a milder form of osteopetrosis compared to Ostm1, therefore suggesting that Ostm1 may have additional functions. The present model defined Ostm1 as an essential partner required for ClC-7 stabilization and protection from lysosomal degradation [63]. This complex is also essential for ClC-7 transport to the osteoclast ruffled border [66].

## 5. Ostm1 in Osteoclast Maturation and Activation

To analyze the role of Ostm1 in a cell specific manner, we generated an *Ostm1*^lox^ allele to induce conditional ablation (cKO) of Ostm1 protein in any tissue. As the first human mutation, we described results from *OSTM1* exon 5 skipping, loxP sites flanking exon 5 were introduced in the mouse locus to mimic the human mutation [67]. We validated functionality of the floxed allele to reproduce a similar osteopetrotic *gl* phenotype by crossing *Ostm1*^lox/lox^ mice with systemic deletion via Meox-Cre^+^ deleter transgenic line [68]. Accordingly, all *Ostm1*^lox/lox^ Meox-Cre^+^ progenies develop severe osteopetrosis and die ~3 weeks after birth. Thus, we generated the first engineered *Ostm1* cKO mouse model.

Subsequently, cKO of *Ostm1* was induced in mature osteoclasts with the Cathepsin K (Ctsk-Cre) deleter transgenic line [69] to generate *Ostm1*Δ^exon5^ Ctsk-Cre^+^ homozygous mice. An in-frame exon 5 deletion was subsequently confirmed by sequencing and the recombination level in mature *Ostm1*^lox/lox^ Cre^+^ osteoclasts approached 100%. *Ostm1*^lox/lox^ Ctsk-Cre^+^ mice develop severe osteopetrosis similar to the *gl*/*gl* phenotype with a short lifespan of ~3 weeks [67]. We also generated compound heterozygous *Ostm1*Δ^exon5/+^ Ctsk-Cre^+^ mice that display a normal phenotype, excluding a dominant negative effect for the truncated/secreted Ostm1 protein as proposed in cell culture systems [59]. Our results are in accordance with heterozygous *OSTM1* patients that are asymptomatic.

Analogous to systemic *Ostm1* loss of function, we first showed that conditional ablation of *Ostm1* in osteoclasts does not affect osteoclast differentiation but results in formation of numerous oversized multinucleated osteoclasts in vivo and ex vivo [67] similar to mice deficient in ClC-7 [70]. Normally, upon recruitment and attachment to bone matrix in vivo, committed mononuclear pre-osteoclasts undergo a process of fusion that gives rise to multinucleated mature osteoclasts [71,72,73]. Expression of the main fusogenic genes dendrocyte expressed seven trans-membrane protein (*DC-Stamp*), osteoclast stimulatory trans-membrane protein (*OC-Stamp; Tm7sf4*), and the d2 isoform of vacuolar ATPase V0 domain (*Atp6v0d2*) is essential in this mechanism [74,75,76]. The disruption of these genes inhibits the osteoclast fusion processes, blocks osteoclast maturation, and prevents bone resorption [77]. The role of the trans-membrane protein DC-Stamp in osteoclast fusion was demonstrated in *DC-Stamp* null mice that exhibit formation of only mononucleated pre-osteoclasts less capable to resorbing bone matrix, whereas transgenic mice overexpressing DC-Stamp accumulate osteoclasts of greater size with accelerated fusion [78]. Similarly, inactivation of the *Atp6v0d2* gene led to reduced osteoclast fusion and defective bone resorption [76]. Consistent with osteoclast multi-nucleation stimulation due to the absence of *Ostm1*, a significant increase in transcription upregulation of these fusogenic genes was quantified in the oversized multinucleated cKO osteoclasts. This result was further substantiated with the upregulated expression and nuclear relocalization of the transcription factor Nfatc1, the upstream master regulator that controls expression of these target genes [78]. Together, these data demonstrate that Ostm1 is a negative regulator of the Nfatc1 pathway essential for osteoclast multinucleation [67]. 

Secondly, we demonstrated in vitro that cKO osteoclasts isolated from *Ostm1*^lox/lox^ Ctsk-Cre^+^ mice were larger in size and were able to form a peripheral actin ring and podosomes, critical for osteoclast tight attachment to bone matrix. However, these cKO cells are defective in bone resorption similar to osteoclasts with complete loss of *Ostm1* in *gl*/*gl* mice. In normal conditions, upon bone matrix interaction, polarization of multinucleated osteoclasts induces cytoskeletal rearrangements and formation of a ruffled border essential for proper bone resorption [79]. This structure is highly dependent on osteoclast secretory lysosomes that traffic and fuse to the plasma membrane to create an acidic (pH ~4.5) resorption lacunae [14,20,80]. Interestingly, autophagy proteins LC3-II, Atg5, Atg7, and Atg4B were also reported to participate in the formation of this cellular structure [81,82] and deficiency in such proteins impaired ruffled border development, but the underlying process is still undefined. The resorption lacunae acidification relies on the proton pump (H^+^/V-ATPase) and chloride transporter ClC-7 that secrete hydrogen and chloride ions into the lacunae [83]. The importance of H^+^/V-ATPase in mouse and humans was characterized by mutations in the a3 (*oc; Tcirg1)*, Atp6v1c1 and Atp6ap1 (Ac45) enzyme subunits that result in non-functional osteoclasts [84,85,86,87]. Likewise, ClC-7 deficiency in mice and in humans causes osteopetrosis with variable severity [64,88].

**Figure 3:**
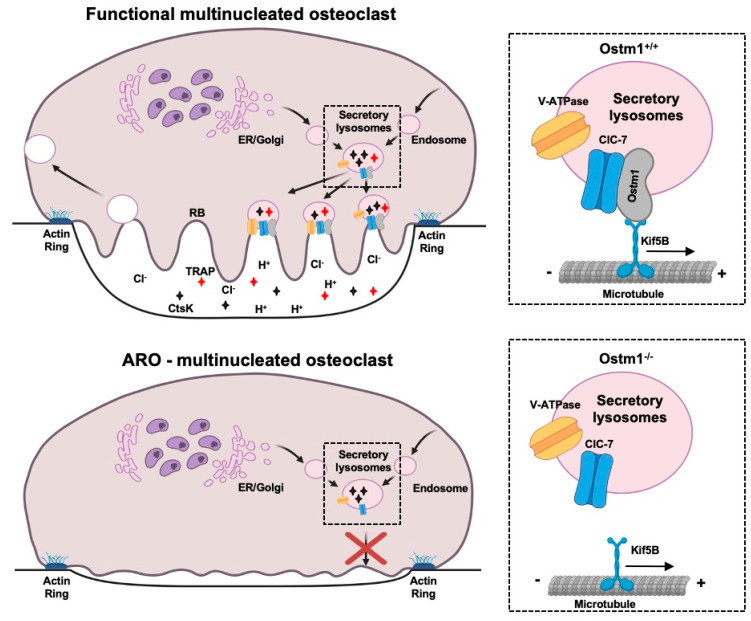
Proposed working model of Ostm1-mediated osteoclast dysfunction leading to autosomal recessive osteopetrosis. RB: Ruffled border; ER: Endoplasmic Reticulum In ARO osteoclast, impaired secretory lysosome trafficking results in lack of ruffled border formation and ineffective bone matrix resorption.

In *Ostm1* cKO osteoclasts, we also demonstrated that the acidic luminal pH of the secretory lysosomes, essential for their function, was not altered [67] as described in *gl*/*gl* fibroblasts and neurons [63]. However, lysosomes showed a more disperse repartition and localization in resorbing cKO osteoclasts [67]. This result demonstrated that loss of Ostm1 directly affects intracellular dispersion and relocalization of the acidic endolysosomal compartment possibly through an interaction with the motor protein Kif5B, a partner of Ostm1 [58] (Figure 3).

Further evidence for a trafficking defect in absence of *Ostm1* was obtained by analysis of the *Acp5* (Trap) and *Ctsk* genes expression levels and proteins release. We showed that transcription levels of *Acp5* and *Ctsk* were significantly enhanced in *Ostm1* cKO osteoclasts. This response was consistent with concomitant upregulation of the Nfatc1 transcriptional factor expression since both *Acp5* and *Ctsk* gene promotors are targets of Nfatc1. We then determined if the loss of *Ostm1* can affect osteoclast protease release through biochemical quantification of extracellular levels of Trap and Ctsk enzymes. In *Ostm1* cKO osteoclast cultures, Trap release was strongly reduced whereas the secreted Ctsk protein was undetectable. Impaired release of these proteases confirms that Ostm1 plays a role in osteoclast secretory lysosome trafficking and possibly in exocytosis (Figure 3). Additional support for a major role of Ostm1 in endosome/lysosome trafficking was described in the context of B cell lymphoma drug sensitivity [89] as well as in lysosome formation in osteoclasts [90]. These results demonstrate an osteoclast cell-intrinsic role for *Ostm1* as a positive regulator of secretory lysosome dispersion, independent of other bone cells such as osteoblasts.

## 6. Ostm1 in Non-Bone Tissues

The *gl*/*gl* hematopoietic multi-lineage defects were functionally rescued by enabling Ostm1 cDNA expression under the control of the PU.1 transcription factor regulatory sequences [44]. This was accomplished by replacing the coding sequence of PU.1 with that of Ostm1 using homologous recombination in a PU.1 BAC. Several PU.1-Ostm1 BAC transgenic lines were produced with no phenotype and few were successively crossed to *gl*/+ mice to obtain PU.1-Ostm1-*gl*/*gl* homozygous animals. All PU.1-Ostm1 BAC *gl*/*gl* progenies from one line were rescued of *gl*/*gl* osteoclast defects, including osteopetrosis, but also of the altered myeloid and lymphoid lineages. This study provided evidence that *Ostm1* is required independently for osteoclast and hematopoietic lineages.

However, PU.1-Ostm1-*gl*/*gl* transgenic mice still have a limited extended lifespan of ~7–8 weeks and still undergo premature death [65]. We investigated the cause of death of PU.1-Ostm1-*gl*/*gl* BAC mice and we first determined that *Ostm1* was highly expressed in neurons and to a lesser extent in microglia and astrocytes. Consistent with the neurological disorders observed in some patients [52], the PU.1-Ostm1-*gl*/*gl* BAC mice develop brain inflammation with astrogliosis and microgliosis. Simultaneously, a rapidly progressive neurodegeneration affects all parts of the brain including cortex, hippocampus, cerebellum, and retinal degeneration that was associated with loss of photoreceptors [65]. This latter phenotype was most likely not a secondary effect due to excess bone accumulation and cranial nerve compression described in some osteopetrosis forms [55] but rather suggests an intrinsic neuronal role of *Ostm1* that will be further analyzed. 

The massive neuronal cell loss in PU.1-Ostm1-*gl*/*gl* BAC mice progressed swiftly from 3 to 7–8 weeks and cytosolic ubiquitin accumulation in osmophilic inclusions in neurons, suggested a storage-autophagy disorder. Demyelination was also occurring as previously observed in *Ostm1* null *gl*/*gl* mice [53,91]. Through functional rescue using a series of targeted Ostm1 transgenic mice to individual brain cell-types, we demonstrated that neuronal death in these mice was specifically due to Ostm1 loss in neurons, excluding a direct implication of astrocyte or microglia cells. These mice represent the first in vivo model to analyze the neurological function of Ostm1.

Our cellular analysis in these mice unraveled a marked accumulation of autophagosomes in neurons from the cortex and hippocampus, indicative of an impaired autophagy mechanism. This phenotype results in axonal swelling consistent with a trafficking defect [65]. In this PU.1-Ostm1-*gl*/*gl* BAC model, the neuronal pathology features an autophagy mechanism independent of Beclin1 signaling, but reliant on downregulation of the mTOR (Mechanistic Target of Rapamycin) pathway and downstream targets [65]. These phenotypic studies on the loss of *Ostm1* in the central nervous system (CNS) showed some similarities with known lysosomal storage diseases like Parkinson’s and Alzheimer’s [92,93,94]. More importantly, these findings were correlated with neuropathologic defects observed in *OSTM1* patients further supporting an essential primary role of *Ostm1* in the CNS, independent of the hematopoietic lineage [52].

## 7. OSTM1 and Human Osteopetrotic Patients

*OSTM1* mutations are responsible for the most severe form of infantile type 3 autosomal recessive osteopetrosis with neuropathy (OMIM no. 259720) [52,95,96]. Presently, very few patients with OSTM1 loss of function mutations have been characterized (which include splice site variants, frameshift, and nonsense) and they represent around 5% of spontaneous human ARO [49,51,53,97,98]. Additional patients with *OSTM1* micro-deletions defined a new class of mutations to be considered in diagnostic screening [99]. In parallel, new available molecular technologies such as high-throughput exome sequencing greatly facilitate identification and characterization of new mutations in carrier families [100]. 

Until now, allogeneic hematopoietic stem cell transplantation (HSCT) is the only curative treatment for ARO but the success rate is not optimal with engraftment being a limiting factor and overall outcomes remain disappointing [101,102,103,104]. However, significant improvements in transplantation success rates were obtained with safer regimens and reduced drug conditioning [105] as well as the use of non-invasive magnetic resonance imaging (MRI) of post-transplantation skeletal remodeling [106].

Early in utero interventions were also successfully designed to restore osteoclast activity in *oc (Tcirg1^−/−^)* mice [107]. Alternatively, a novel protocol tested in mice consists in transfusion of monocytic cells that can rescue bone marrow development in an early onset of osteopetrosis in the absence of HSCT [108]. As validation of this concept, microglial engraftment through single in utero transplant in the mouse can improve some of the brain phenotype described in lysosomal storage disease [109] and could possibly alleviate neuronal defects due to osteopetrosis. In fact, based on the implication of *Ostm1* in neuronal homeostasis, curative treatment of *OSTM1* patients is still challenging and consensus guidelines are being established [110]. Despite successful HSCT, neuronal pathology progresses in some *OSTM1* patients [111]. However, the possible use of less invasive monocytic transfusions can give promise of less painful, more humane, and versatile therapies for *OSTM1* patients. 

## 8. Concluding Remarks

Our successful quest to understand the mouse grey-lethal osteopetrotic mutation was a long journey but it has allowed us to characterize the previously unknown *Ostm1* locus responsible for the most severe form of osteopetrosis in mice and humans. All of these studies were made possible through generation of mouse models as well as molecular and cellular protocols to analyze osteoclast phenotypes. In osteoclasts, *Ostm1* is a negative modulator of cell multi-nucleation, an essential step toward cell maturation. In osteoclast activation, Ostm1 along with specific partners is a positive regulator of intracellular trafficking of secretory lysosomes responsible for ruffled border formation, extracellular acidification, and bone matrix degradation. Therefore, *Ostm1* expression in mature osteoclasts is absolutely required to prevent osteopetrosis. For *OSTM1* patients, major progress has been made by the design of prenatal screens in carrier families, however more studies are needed to develop efficient curative treatments since these patients can frequently relapse independently from the hematopoietic cell lineage itself. The characterization of the molecular mechanisms of *Ostm1* was of fundamental importance for our understanding of osteoclast biology, but also of high clinical relevance for bone diseases like osteopetrosis. Further studies in mice are still required, including discovery of additional Ostm1 partners to get insights in the complex bone cell crosstalk responsible for maintenance of bone matrix homeostasis. The osteoclast remains a fascinating cell to study and a better knowledge of its biology and multiple cellular interactions will characterize novel therapeutic targets for major bone diseases.
